# A simplified Bcl-2 network model reveals quantitative determinants of cell-to-cell variation in sensitivity to anti-mitotic chemotherapeutics

**DOI:** 10.1038/srep36585

**Published:** 2016-11-04

**Authors:** Hao Yuan Kueh, Yanting Zhu, Jue Shi

**Affiliations:** 1 Division of Biology, California Institute of Technology, Pasadena, CA 91125, USA; 2 Center for Quantitative Systems Biology, Hong Kong Baptist University, Hong Kong, China; 3 Department of Physics and Department of Biology, Hong Kong Baptist University, Hong Kong, China

## Abstract

Anti-mitotic drugs constitute a major class of cytotoxic chemotherapeutics used in the clinic, killing cancer cells by inducing prolonged mitotic arrest that activates intrinsic apoptosis. Anti-mitotics-induced apoptosis is known to involve degradation of anti-apoptotic Bcl-2 proteins during mitotic arrest; however, it remains unclear how this mechanism accounts for significant heterogeneity observed in the cell death responses both within and between cancer cell types. To unravel quantitative determinants underlying variability in anti-mitotic drug response, we constructed a single-cell dynamical Bcl-2 network model describing cell death control during mitotic arrest, and constrained the model using experimental data from four representative cancer cell lines. The modeling analysis revealed that, given a variable, slowly accumulating pro-apoptotic signal arising from anti-apoptotic protein degradation, generation of a switch-like apoptotic response requires formation of pro-apoptotic Bak complexes with hundreds of subunits, suggesting a crucial role for high-order cooperativity. Moreover, we found that cell-type variation in susceptibility to drug-induced mitotic death arises primarily from differential expression of the anti-apoptotic proteins Bcl-xL and Mcl-1 relative to Bak. The dependence of anti-mitotic drug response on Bcl-xL and Mcl-1 that we derived from the modeling analysis provides a quantitative measure to predict sensitivity of distinct cancer cells to anti-mitotic drug treatment.

Anti-mitotic drugs, one of the most commonly used anticancer chemotherapeutics in the clinic, inhibit cancer cell growth mainly by disrupting the formation of bipolar spindle in mitosis, subsequently arresting cells in prolonged mitotic arrest, from which cells may die or slip out to an abnormal G1 state^1^. Current anti-mitotic drugs include the classic microtubule-targeting drugs, such as taxanes (paclitaxel and its derivatives) and vinca alkaloids (vinblastine, vincristine and their derivatives), as well as the new, more spindle-specific drugs, such as inhibitors of Kinesin-5 (aka KSP, Eg5, KIF11), Aurora-A, Aurora-B and Polo-1 kinases^2^,^3^,^4^. Although widely used, in particular taxanes for treating solid tumor, anti-mitotics are ineffective for many types of cancer; and sensitive cancers tend to acquire resistance. In order to improve the effectiveness of current anti-mitotic therapy, a better understanding of the quantitative mechanisms underlying the strong cell-to-cell variation in anti-mitotic drug response is clearly needed, and shall provide the molecular basis to develop diagnostic measure to identify sub-populations of patients that may respond well to anti-mitotics as well as for designing new combinatorial therapies.

While anti-mitotics at sufficiently high concentration can induce mitotic arrest in all proliferating cells, sensitivity and kinetics to induction of cell death during or after the arrest is highly variable across different cancer cell types in both cultured human cells^5^ and syngeneic mouse tumors^6^. In other words, the most variable point of anti-mitotic drug effect both within and between cancer types is in activating cell death, which is known to be mostly mediated by the intrinsic, or mitochondrial, apoptosis pathway^7^,^8^. One prominent characteristic of anti-mitotics triggered apoptosis is that cells arrest for many hours in mitosis before apoptosis is initiated; and the long delay from mitotic entry to apoptosis is highly variable in individual cells. We have previously investigated the slowly accumulating pro-apoptotic signal in prolonged mitotic arrest and identified depletion of Mcl-1, due to transcriptional silence, was one key pro-apoptotic trigger to activate mitotic death^9^. Moreover, by imaging a live-cell fluorescent reporter of mitochondrial outer membrane permeabilization (MOMP)^10^, the committed step of intrinsic apoptosis, we have shown that MOMP preceded nearly all cell death activated during mitotic arrest, and was rapid and switch-like, completing within minutes. MOMP is known to be regulated by Bcl-2 family proteins, such as Mcl-1; however, it is unresolved how a long, gradual pro-apoptotic signal from Mcl-1 depletion, which decays exponentially in the time scale of hours, may give rise to a sharp, all-or-none induction of apoptosis within minutes. In this study, we will perform both analytical and numerical analysis of the dynamics of a simplified Bcl-2 network to elucidate the quantitative mechanism that links a gradual, exponential signal to MOMP and the rapid MOMP induction across distinct timescales.

The other key question that we will address in this computational study is the quantitative origins of cell-to-cell variation in both sensitivity and kinetics to apoptosis during anti-mitotics-induced mitotic arrest. We chose to focus on analyzing mitotic death control, but not death after slippage, as it is the most variable point in the response to anti-mitotic drugs. Mcl-1 is known to be depleted to similar final levels in both apoptosis-sensitive and -resistant cell lines, thus loss of Mcl-1 alone cannot account for the apoptosis regulation during mitotic arrest. Based on results from gene knockdown by RNA interference (RNAi), we previously pinpointed Bcl-xL, but not Bcl-2, Bcl-w or pro-apoptotic BH3 proteins, as the other key regulator of apoptosis in mitotic arrest^9^. Variation in expression levels of Mcl-1 and Bcl-xL largely determine variability in sensitivity to mitotic death induced by anti-mitotics, such as paxlitaxel and Kinesin-5 inhibitor, across different cultured cancer cell lines. That is, the threshold for triggering cell death during mitotic arrest is mainly determined by basal expression levels of Mcl-1 and Bcl-xL. However, in order to employ Mcl-1 and Bcl-xL as diagnostic markers to predict anti-mitotic drug response in patients with distinct cancer types and heterogeneous tumor mass, we need to establish the quantitative, beyond qualitative, dependence of anti-mitotic drug response on Mcl-1 and Bcl-xL expression levels and their depletion kinetics, as well as determine to what extent the variation in the above dynamic parameters impacts the degree of variability in drug response both between cancer cell types (i.e., inter-cell line variability) and within a cancer type (intra-cell line variability). Therefore, in this study we conducted computational simulation of the simplified Bcl-2 network model for mitotic death control to analyze cell-to-cell variation at the single cell level, profiled the parameter space of levels and kinetics of Mcl-1 and Bcl-xL, and then derived the quantitative dependence of individual cell response to mitotic death induced by anti-mitotic drugs.

## Results

### Defining Bcl-2 network components for mitotic death control

**Figure 1:**
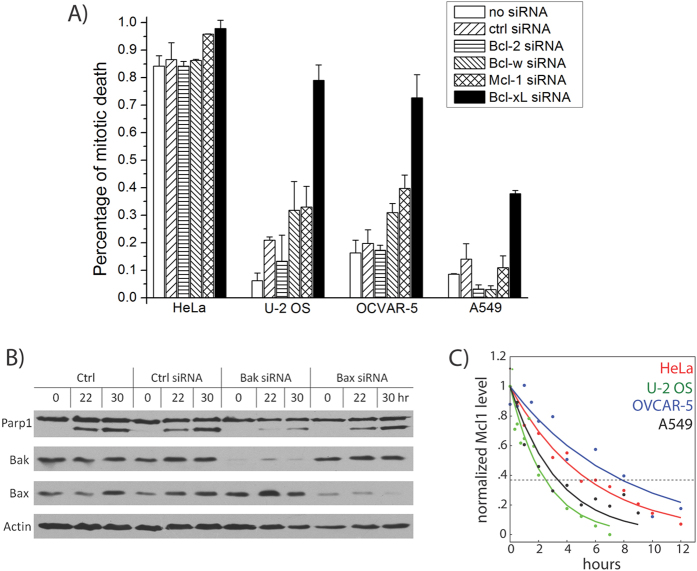
Defining key Bcl-2 network components for mitotic death control in different cell lines. (**A**) Inhibitory Bcl-2 family proteins were knocked down using siRNA treatment in the indicated cell lines. Cells were arrested in mitosis by treatment with an anti-mitotic drug, Kinesin-5 inhibitor (K5I), and dead cell percentages were then scored after 48 hours of drug treatment using time-lapse microscopy. Data were re-plotted from ref. 9. (**B**) Pro-apoptotic Bcl-2 family proteins Bax and Bak were knocked down in HeLa cells, using RNAi. Cells were then arrested in mitosis by K5I, and analyzed using western blotting for Parp1 cleavage, an indicator of apoptotic death. Results showed that knockdown of Bak, but not Bax, attenuates mitotic cell death. (**C**) Cells were arrested in mitosis using K5I treatment, and analyzed for Mcl-1 levels using quantitative western blotting at the indicated time points. Plot shows Mcl-1 levels over time, with curves indicating best fits to a single exponential decay in the form of *f*(*t*) = *A*_0_ exp (−t/τ). The decay time constants *τ* derived here are subsequently used to constrain modeling fits.

We had previously identified the key Bcl-2 family proteins responsible for mitotic death control by studying four representative cancer cell lines: HeLa, U-2 OS, OVCAR-5 and A549^9^. These lines were chosen, as they cover a wide spectrum of sensitivity to mitotic death induced by anti-mitotic drugs (e.g., paclitaxel and Kinesin-5 inhibitor), based on drug response profiling experiments^5^. By knocking down candidate Bcl-2 family proteins using siRNA treatment and then determining the resulting effects on mitotic death, we found that knockdown of the anti-apoptotic proteins Bcl-xL and, to a lesser extent, Mcl-1, enhanced cell death during drug-induced mitotic arrest (Fig. 1A) in all four cell lines, in particular the three resistant cell lines (U-2 OS, OVCAR-5 and A549), albeit to varying degrees. In contrast, knockdown of Bcl-2 or Bcl-w showed mostly minimal effect across the cell lines, suggesting that they play largely negligible roles in regulating mitotic death. Together with data showing that activator BH3 proteins, such as Bim and tBid, or up-regulation of sensitizer BH3-only proteins are not required for mitotic cell death, we concluded that Mcl-1 and Bcl-xL are the key negative regulators of cell death acting during prolonged mitotic arrest^5^,^9^.

Mcl-1 and Bcl-xL are known to inhibit cell death by sequestering the pro-apoptotic proteins Bak and Bax, which oligomerize to form pores in mitochondrial membrane to trigger MOMP. To determine whether Bak and/or Bax are involved in mitotic death control, we knocked down both proteins by RNAi in HeLa (the cell line that is highly sensitive to mitotic death), and determined the resultant effects on cell death by measuring Parp1 cleavage (Fig. 1B). We found that loss of Bak, but not Bax, significantly attenuated the extent of mitotic death (Fig. 1C), suggesting that Mcl-1 and Bcl-xL protect cells from mitotic death primarily through inhibitory interactions with Bak during mitotic arrest.

Transcription and translation are attenuated during prolonged mitotic arrest^11^,^12^,^13^, and we proposed that such mitotic silencing of gene expression selectively depletes unstable anti-apoptotic proteins to trigger cell death. Consistent with this idea, we found that Mcl-1 protein levels decreased steadily upon mitotic arrest in all four cell lines, with half-lives shorter than the observed timescales of mitotic cell death (τ_d_ ~ 3–8 hrs, Fig. 1C). In further agreement with this model, Bcl-xL has a measured protein half-life comparable to the timescales of mitotic death induction (τ_d_ ~ 10 hours^14^), whereas Bak is significantly more stable (τ_d_ ~ 170 hours^15^). Taken together, these results point to a mechanism, where degradation of Mcl-1 and Bcl-xL protein during mitotic arrest relieves Bak from inhibition, allowing it to form pores that permeabilize the outer mitochondrial membrane and trigger cell death.

### Modeling of the simplified Bcl-2 network identifies a requirement for high-order cooperativity in Bak pore formation

**Figure 2:**
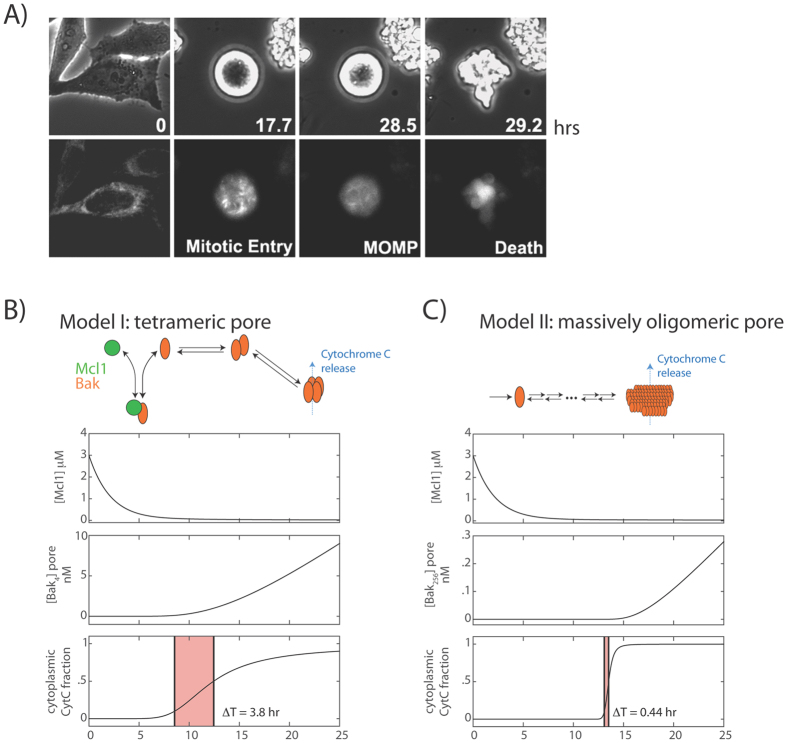
Switch-like apoptosis induction during mitotic arrest requires large oligomeric mitochondrial membrane pores. (**A**) Kinetics of MOMP in HeLa cells arrested in mitosis from time-lapse imaging. To induce mitotic arrest, cells were treated with 1 μM K5I at t = 0 hrs. Still frames show phase-contrast images (top), and fluorescence images of the MOMP reporter, IMS-RP (bottom), which consists of monomeric red fluorescent protein targeted to the inter-membrane space of mitochondria by fusion to the leader peptide of SMAC^10^. In this example, the cell rounded up to enter mitosis at t = 17.7 hrs after drug addition, underwent MOMP at t = 28.5 hrs, indicated by a change from punctate to smooth distribution in fluorescence, and started to bleb and lyse about 20 minutes later (t = 29.2 hr). (**B,C**) Candidate models for explaining the observed kinetics of apoptosis induction, involving either the formation of a tetrameric Bak pore (**B**), or a massively oligomeric Bak pore with hundreds of subunits (**C**). Plots show simulated time evolution of free Mcl-1 concentrations (top), mitochondrial Bak pore concentrations (middle), and fraction of cytoplasmic CytC (bottom), an indicator of death induction. Red shaded area indicates the duration of death induction *ΔT*, defined as the time required for the cytoplasmic CytC fraction to increase from 0.1 to 0.5. The results show that the massively oligomeric pore model (**C**), but not the tetrameric pore model (**B**), can sufficiently account for both the delayed and switch-like kinetics of apoptosis induction observed experimentally.

After cells enter mitotic arrest upon anti-mitotic drug treatment, they typically persist for many hours in a live mitotic state before undergoing apoptosis (Fig. 2A, see also^16^). The switch from a live to dead state with a permeable outer mitochondrial membrane occurs rapidly, reaching completion in minutes (Fig. 2A and Supplementary Movie SM1). Here, we first determined whether the ‘inhibitor decay mechanism’ described above accounts for kinetic properties of such a cell death switch. Specifically, we used mathematical modeling to determine what Bcl-2 network reaction schemes can give rise to: 1) long time delays preceding apoptosis induction, typically 10 hours or more; and 2) rapid MOMP execution, within 20 minutes or less. Our analysis combines numerical simulations of ordinary differential simulations (Fig. 2B,C) with analytical approaches (see Materials and Methods), which generate insights that hold, independent of the exact parameter choices or specific network architecture.

Based on existing biochemical evidence^17^, we first constructed a model, where Bak monomer either binds Mcl-1 to form an inactive complex, or undergoes two sequential dimerization reactions to form tetramer pores on the mitochondrial membrane, resulting in cytochrome C (CytC) translocation from mitochondria to cytoplasm (Model I, Fig. 2B). The total Bak protein level remains constant due to its high stability, whereas Mcl-1 level decreases over time with first-order kinetics. From numerical simulations (see Materials and Methods, and Table 1), we found that this model can give rise to delayed apoptosis induction after Mcl-1 decay, as intuitively expected (Fig. 2B). After the onset of mitotic arrest (t = 0 hrs), the concentration of free Mcl-1 decreases over time, causing an eventual increase in the concentration of Bak tetramer and CytC in the cytoplasm. However, while the simulation results account for the delayed timing of apoptosis switch (~10 hours after mitotic arrest), they were not able to recapitulate the sharp switch itself. With the simulations, we found that the switch timing *ΔT*, taken to be the time for the fraction of CytC in the cytoplasm to increase from 0.1 to 0.5, was 3.8 hours, much longer than the switching time of <20 minutes observed experimentally (Fig. 2A). This suggests that this Bak tetramer model, while intuitively appealing, is not sufficient to account for the switch-like properties of the apoptotic response in mitotically arrested cells.

In signaling systems, switch-like behavior can emerge from induced clustering of membrane-associated receptor proteins into large complexes^18^,^19^,^20^. Motivated by these examples, we examined an alternative possibility, where Bak oligomerizes to form large pores containing hundreds of subunits (Model II). We analyzed an example reaction scheme, where Bak monomer undergoes multiple sequential dimerization reactions to generate an active pore consisting of 256 subunits. Nonetheless, the modeling results, as shown below, do not depend on the exact number of subunits of the active pore, or on the exact reaction scheme for generating this active pore. As before, Mcl-1 forms an inactive complex with Bak, and degrades with first-order kinetics. From numerical simulations (see Materials and Methods, and Table 1), we see that this model not only gave a delayed induction of Bak pores upon Mcl-1 protein decay (Fig. 2C, top and middle), but also generated a much sharper MOMP reaction compared to the tetramer pore model (*ΔT* = 0.44 hours), in closer agreement with experimental observations (Fig. 2A). These results suggest that a switch-like apoptotic response requires high-order cooperativity from the formation of mitochondrial membrane pores consisting of hundreds of Bak/Bax subunits. To establish the generality of this result, we solved the models analytically to obtain *T*_*c*_, the duration of the time delay before apoptosis, and *ΔT*, the induction time of apoptosis. For both models, we find that:


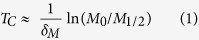


where *δ*_*M*_ gives the first-order rate of Mcl-1 degradation, *M*_0_ represents the initial Mcl-1 level, and *M*_1/2_ (<*M*_*0*_) gives the critical Mcl-1 level for apoptosis induction, and


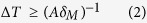


where *A* represents the number of subunits making up the active pore (*A* = 4 for Model I, and *A* = 256 for Model II). Evidently, when pore size is large, *ΔT* is small, corresponding to sharp MOMP induction. This result makes intuitive sense. As large pores require more Bak freed from sequestration by Mcl-1, Mcl-1 has to be depleted to a lower level than that for small pores in order to trigger the onset of MOMP. Depletion to lower Mcl-1 level obviously takes longer time, resulting in a longer delayed time to initiate MOMP and a sharper switch response.

Quantitatively, in order to generate a sharp switch of MOMP after a long delay, we must fulfill the requirement that Δ*T* ≪ *T*_C_, which requires:


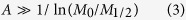


The logarithm of the fold change in free Mcl-1 levels on the right hand side of this expression is of order unity; therefore, consistent with our simulation results, this analytical result shows that sharp switching can occur only when the number of subunits in the active Bak pore, *A,* is much greater than one. Indeed, recent imaging studies have found the Bax and Bak structures on the mitochondrial membranes of apoptotic cells are estimated to contain hundreds or even thousands of subunits^21^,^22^,^23^, consistent with this notion. Our modeling results, taken together with these experimental studies, argue that sharp apoptotic switching cannot emerge simply through formation of small pores with <10 subunits, but requires highly cooperative assembly of large mitochondrial membrane pores with hundreds or even thousands of Bak/Bax subunits.

### Bcl-2 network modeling recapitulates and quantifies single cell variability in apoptotic responses for different cancer cell lines during mitotic arrest

**Figure 3:**
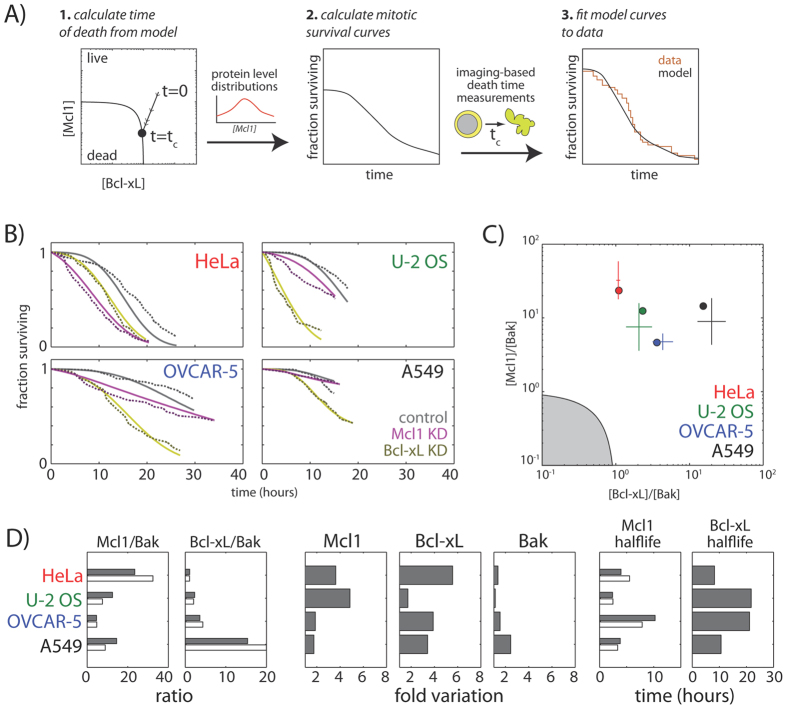
Quantitative insights into cell-to-cell variation in apoptotic response from modeling and computational analysis. (**A**) Procedure for fitting model-derived survival curves to experimental data. Mitotic survival curves derived from dynamical models were fit to experimental data for HeLa, U-2 OS, OVCAR-5 and A549, obtained under both control conditions and upon Mcl-1 or Bcl-xL knockdown. (**B**) Fraction of mitotically-arrested cells surviving over time under control conditions (grey), upon Mcl-1 knockdown (purple), and upon Bcl-xL knockdown (gold). Dotted lines represent experimental data, and solid lines represent best fits to the model. (**C**) Mcl-1/Bak versus Bcl-xL/Bak ratios for best model fits to the four cell lines (solid circles). Crosses represent relative ratios between the cell lines measured by western blotting (mean and standard deviation, N = 3). Gray shaded area represents forbidden region where Mcl-1 and Bcl-xL levels are insufficient to maintain normal cell survival. (**D**) Histograms showing best fit model parameters (solid bars), along with experimentally measured values (hollow bars). Fold variation shows cell-to-cell variability in protein levels, as modeled using a log-normal distribution. Here, measured Mcl-1 and Bcl-xL to Bak ratios are scaled by constant factor to allow comparison with model parameters.

Given that the simplified Bcl-2 network model (i.e., model II) accounts for the basic kinetic properties of mitotic death response, we next investigate whether it can be employed to examine the variable single-cell death responses in different cancer cell types during mitotic arrest, e.g., the four representative cell lines HeLa, U-2 OS, OVCAR-5 and A549 (Fig. 1A). Previous studies have implicated cell-to-cell differences in protein concentrations as a cause of non-genetic variability in apoptosis timing in human cell lines^24^. Therefore, we combined our kinetic model with probability distributions of apoptotic protein abundances in single cells to obtain predicted mitotic survival curves, which give the fraction of surviving mitotic cells as a function of the duration of mitotic arrest (Fig. 3A, center; see also Materials and Methods). In these single cell simulations, we explicitly included Bcl-xL level, in line with experimental results (Fig. 1A, see Materials and Methods), and also included an implicit requirement for switch-like apoptosis induction by large Bak oligomers, in line with insights from kinetic modeling (Fig. 2). We then used least-squares fitting to fit these survival curves to those acquired experimentally for the four cell lines, both under control conditions and upon knockdown of either Mcl-1 or Bcl-xL. Fits were constrained using measured Mcl-1 protein half-life data (Fig. 1C) and quantitative protein level measurements from western blotting (means and standard deviations shown in Fig. 3C as crosses), which constrained the means of the single-cell protein level distributions.

From model fitting and analysis, we found that this simple model for apoptosis induction recapitulates the following key properties of the variable apoptotic response in the four cell lines that we previously studied (Fig. 3B): (1) cell-to-cell variability in apoptotic death kinetics observed within individual cell lines, both under control conditions and upon Mcl-1 or Bcl-xL knockdown (Fig. 3B); (2) differences in apoptotic death timing between different cell lines, and, in particular, the differential sensitivities of the four cell lines to knockdown of Mcl-1 or Bcl-xL (Fig. 3B); (3) differences in the ratios of Mcl-1, Bcl-xL and Bak basal expression levels across the four cell lines (Fig. 3C,D); and (4) differences in Mcl-1 protein half-lives across the four cell lines (Fig. 1D). In addition to accounting for these experimental measurements, our model also recapitulates the published decay rates for Bcl-xL^14^ (τ ~ 10–20 hrs, Fig. 3C), which were not used to constrain our model. While small differences in the shapes of the mitotic survival curves from experimental data were observed (Fig. 3B), the fairly close agreement of the models with data on multiple levels allowed us to derive quantitative insights and generate hypotheses beyond the experimental findings. Specifically, we made two major observations and predictions as follow:

Firstly, variability in the levels of anti-apoptotic regulators underlies heterogeneity in death responses within cell lines (i.e., intra-cell line variation). In all four cell lines examined, there was considerable cell-to-cell variability in the timing of apoptosis induction within the cell line, with some cells dying shortly after mitotic arrest, and others persisting for over ten hours before dying (Fig. 3B). This intra-cell line heterogeneity could in principle arise from non-genetic variability in the levels of the anti-apoptotic regulators Mcl-1 or Bcl-xL, or in the level of the pro-apoptosis regulator Bak. Our modeling fits showed considerable cell-to-cell variation in the expression of anti-apoptotic regulators, with cells showing over three-fold variation in the levels of Mcl-1 (U-2 OS), Bcl-xL (Ovcar-5 and A549), or both proteins (HeLa). In contrast, the variation in the levels of the pro-apoptotic protein Bak was considerably smaller in all of the cell lines examined. These observations suggest that cells of the same genetic background may tightly regulate their expression levels of pro-apoptotic proteins, and generate variable death responses primarily through heterogeneous expression of anti-apoptotic proteins.

Secondly, different average levels of apoptotic regulatory proteins underlie the differential susceptibility of cell lines to mitotic death (i.e., inter-cell line variation). The inter-cell line variability is obviously much stronger than that within the same cell line. The four cell lines also showed considerably differing responses to knockdown of anti-apoptotic proteins. Mcl-1 knockdown greatly accelerated apoptotic death in HeLa cells, but had negligible effects in A549 cells; conversely, removal of Bcl-xL increased both the extent and kinetics of death in U-2 OS, OVCAR-5 and A549 cells, but showed only moderate effects in HeLa cells. These differential susceptibilities could arise from cell-line specific differences in protein expression levels, or differences in their degradation rates during mitotic arrest. Our modeling fits showed that HeLa cells, which showed the greatest sensitivity to Mcl-1 knockdown, had the highest Mcl-1/Bak ratio but the lowest Bcl-xL/Bak ratio out of the cell lines examined (Fig. 3B–D); conversely, U-2 OS, OVCAR-5 and A549 cells, which were more susceptible to Bcl-xL knockdown, but not Mcl-1 knockdown, had comparatively lower Mcl-1/Bak ratios but progressively increasing Bcl-xL/Bak ratios. In contrast, we found no correlation between death sensitivity and the half-lives of Mcl-1 or Bcl-xL (Fig. 3D), even though they showed considerable variation between different cell lines. Instead, these parameters appeared to affect the total duration of mitotic arrest, which was longest in cells with the highest stability of these proteins (i.e., OVCAR-5, Fig. 3B–D). Together, our data illustrate that inter-cell line variability depends on the highly expressed anti-apoptotic proteins, and suggest that ratio of the levels of an anti-apoptotic protein to its pro-apoptotic partner may be used as a useful quantitative predictor of the susceptibility of distinct cancer cell types to mitotic death activated by anti-mitotic drugs.

## Discussion

Building on previous experimental data, we constructed a simplified Bcl-2 network model consisting of three key components, Mcl-1, Bcl-xL and Bak, to elucidate the dynamic control of mitotic cell death induced by anti-mitotic drugs and the associated cell-to-cell variability. Computational analysis of this simple kinetic model not only revealed critical dynamical features of the network, e.g., high-order cooperativity arising from massive Bak oligomerization, but also allowed us to examine and pinpoint specific network parameters that provide a metric to predict the susceptibility of different cancer cells to mitotic death induced by anti-mitotic drugs. We found heterogeneity in mitotic death response within a cancer type can be attributed to mainly variation in Mcl-1 and Bcl-xL expression levels in individual cells, but not Bak, with a 2 to 3-fold difference in expression being sufficient to generate significant variation in drug-induced cell fate, i.e., dead vs. live, in a clonal population with identical genetic background. Response variation between different cancer types is also mainly attributed to variable expression, in this case ratio of Bcl-xL/Bak and Mcl-1/Bak. The extent of mitotic death is particularly dependent on the ratio of Bcl-xL/Bak, as a 2-fold difference in this ratio can already distinguish cancer lines that are resistant (e.g., U-2 OS) and sensitive (e.g., HeLa) to mitotic death. In other words, our results suggest that tumor cells with a Bcl-xL/Bak ratio 2-fold larger than that of HeLa are resistant to mitotic death. Moreover, although there is considerable variation in Mcl-1 and Bcl-xL degradation kinetics between different cancer types, our dynamic modeling analysis showed that such variability mostly affects the timing to mitotic death, but not the extent.

Although the simplified Bcl-2 model for mitotic death control was constructed based on cell culture data, there are *in vivo* data that also support its validity. In a study of mouse xenograft models of non-small lung cancer, Tan *et al*. found that Bcl-xL and Mcl-1 are the key MOMP regulators with respect to paclitaxel responses *in vivo*^25^. Their mouse model data are clearly consistent with our results with cultured cell lines^9^, regarding the key regulatory roles of Bcl-xL and Mcl-1 in regulating anti-mitotics-induced cell death. Therefore, our Bcl-2 network model for cell death control upon anti-mitotic drug treatment is likely applicable for *in vivo* situation. Nonetheless, we note there are data that point to potential difference in the mechanism by which cell death is activated *in vivo* vs. *in vitro* by anti-mitotic drugs. While cell death seen in culture was all preceded by prolonged mitotic arrest, Orth *et al*. found that many tumor cells in a mouse xenograpt model of Fibrosarcoma did not enter or progress through paclitaxel-induced mitotic arrest, even though tumor significantly regressed^26^. The question of whether mitotic arrest is required for anti-mitotics-induced cell death *in vivo*, or paclitaxel can induce cell death in interphase without entry into mitotic arrest, clearly needs further mechanistic investigation. In addition, we also note that a previous study by Topam *et al*. showed that although anti-mitotics-induced cell death in cultured cell line was mainly repressed by Bcl-xL, it also involved up-regulation of pro-apoptotic BH-3 proteins^27^. Their result of BH-3 protein involvement in regulating mitotic death was different from our previous experimental data on a cancer cell line panel^5^,^9^. Such discrepancy again requires further study to elucidate and clarify its mechanistic origin.

Both numerical and analytical results from the simplified Bcl-2 network model identified a critical requirement for high-order cooperativity in the generation of a sharp switch of MOMP. The major pro-apoptotic signals that activate mitotic cell death are Mcl-1 and Bcl-xL degradation, which occur in the time scale of hours following mitotic arrest. We find that this long, exponentially decaying signal can give rise to a sharp mitotic death response that completes in minutes, only if Bak pore complexes on the mitochondrial membrane are very large (with >10^2^ subunits). In cells, assembly of such large protein complexes is typically highly cooperative, as it involves a phase transition process, occurring spontaneously when a critical parameter – in this case free Bak monomer concentration – reaches a threshold value. Such phase transition behavior is known to govern assembly in various biological systems, including cytoskeletal polymers^28^, amyloid fibers^29^, and membrane signaling clusters^30^, and our results indicate that it may also be utilized by pro-apoptotic proteins to generate all-or-none death responses. Indeed, it is been long suggested that the active pores that execute MOMP on apoptotic cells contain hundreds or even thousands of subunits^21^,^22^,^23^, consistent with this idea. Further testing of this hypothesis will require a combination of biochemical approaches, in conjunction with genetic and modeling studies.

In this study, we focused on analyzing the quantitative determinants of mitotic cell death induced by anti-mitotic drugs, as it is the drug mechanism that activates the most rapid cancer cell death and is also the most variable point in anti-mitotic drug response. However, anti-mitotic drugs are known to trigger cell death not only during mitotic arrest but also after mitotic slippage into an abnormal G1 state^5^,^31^. Molecular regulators that control cell death after slippage are distinct from those during mitotic arrest, mainly involving proteins associated with DNA damage response, e.g., the p53 pathway^32^,^33^,^34^. For simulation and fitting of the Bcl-2 network model in this study, we explicitly excluded the slippage process and cell population that have exited the mitotic arrest, so as to examine only dynamic features of cell death control during mitotic arrest (refer to Materials and Methods). Given that cell fate after mitotic slippage is mainly regulated by DNA damage response, the quantitative predictors of death response after slippage can be investigated in further study, using various mathematical models of DNA damage response that are already available in the literature, such as the p53 pathway models.

## Methods

### Cell culture

All cell lines were purchased from American Type Culture Collection (ATCC, USA) and cultured under 37 °C and 5% CO_2_ in appropriate medium supplemented with 10% Fetal Calf Serum (FCS), 100 U/ml penicillin and 100 μg/ml streptomycin. HeLa was maintained in DMEM; U-2 OS was maintained in McCoy’s; OVCAR-5 was maintained in RPMI; and A549 was maintained in F-12K. The anti-mitotic drug, Kinesin-5 inhibitor (EMD534085), was provided by Merck-Serono.

### Time-lapse microscopy

Cells were plated in 35 mm imaging dish (μ-dish, ibidi, Germany) and cultured in phenol red-free CO_2_-independent medium (Invitrogen) supplemented with 10% FCS, 100 U/ml penicillin and 100 μl streptomycin. Cell images were acquired with the Nikon TE2000-PFS inverted microscope enclosed in a humidified chamber maintained at 37 °C. Cells were imaged every 10 minutes using a motorized stage and a 20X objective (NA = 0.95). Images were viewed and analyzed using the MetaMorph software (Molecular Dynamics).

### Gene knockdown by RNA interference (RNAi)

siRNA oligos for knocking down Bak (#J-003305-07) and Bax (#J-003308-12) were purchased from Dharmacon. Dharmacon On-Target plus siControl (#D-001810-01) was used as non-targeting siRNA control. siRNA transfections were performed in HeLa cells using HiPerFect (Qiagen), according to manufacturer’s instructions. Experiments were conducted after 48 hrs of gene silencing.

### Western blot analysis

Cell lysates were obtained using LDS sample buffer (NuPAGE, Invitrogen). Proteins were resolved on 10% or 12% Tris-glycine gels and transferred onto PVDF membranes. Blots were probed with commercial primary antibodies and chemiluminescent detection using ECL-plus (Amersham). Primary antibodies: PARP1 (#9542), Bak (#3814) and Bcl-xL (#2762) were purchased from Cell Signaling; Bax (#sc-493) and Mcl-1 (#sc-819) from Santa Cruz; Anti-actin (#A5316) from Sigma was used as a loading control. For western blot analysis of Mcl-1 half-lives in synchronized mitotic cells, we grew large volume of cells in 25 cm dishes to 90% confluency, and then treated the cells with 1 μM Kinesin-5 inhibitor to induce mitotic arrest. After 3 hours of drug treatment, the mitotic fraction of cells was collected at the indicated time points and lysed using LDS sample buffer for western blot analysis of Mcl-1.

### A simplified Bcl-2 network model of Bak pore formation and apoptosis induction in mitotic arrest

#### Model description

We present here two ordinary differential equation (ODE) models for apoptosis induction, explicitly considering degradation of Mcl-1 protein during mitotic arrest, sequestration of Bak monomers by the anti-apoptotic protein Mcl-1, Bak oligomerization and resultant pore formation, and transport of Cytochrome C across the outer mitochondrial membrane. In Model I (Fig. 2B), the Bak tetramer forms the active mitochondrial membrane pore. This model is described by the following equations:


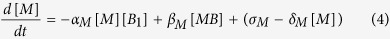



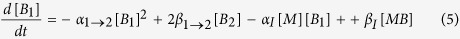



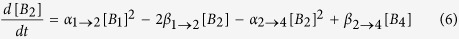



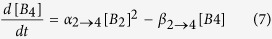


Here, *M* represents Mcl-1; *B*_*n*_ represents the Bak n-mer formed through the indicated oligomerization reactions; *α* and *β* give the rate constants of association and dissociation for the indicated Bak n-mers; *σ*_*M*_ gives the basal Mcl-1 synthesis rate during mitosis; and *δ*_*M*_ gives the first-order rate constant of Mcl-1 degradation during mitotic arrest. Here, we assume that Bak shows negligible degradation over the timescales of mitotic arrest, which is consistent with previous stability measurements of this protein in isotope switching proteomic experiments^15^.

For Model II (Fig. 2C), we assume the active membrane pore is Bak oligomer consisting of 256 subunits, formed through successive dimerization of smaller oligomers, as follows:


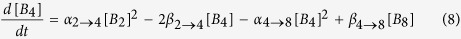



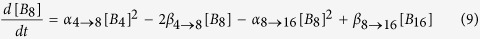



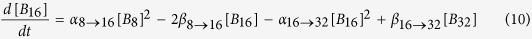



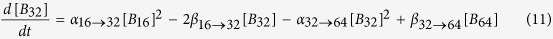



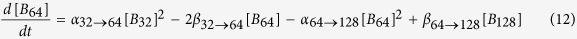



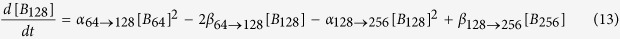



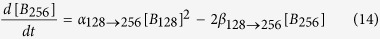


Here, Mcl-1 dynamics, Mcl-1/Bak monomer interactions and the Bak dimerization reaction all remain the same, and are given by (Eqs 4, 5, 6). While this model assumes that the active pore has 256 subunits, our subsequent analysis will show that our conclusions will neither depend on the exact size of the active pore complex, nor on the detailed reaction scheme underlying the generation of the massively oligomeric pore. In both models, the total concentration of Bak is a constant:


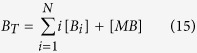


where the summation is performed over all Bak oligomeric species. Finally, for both models, the translocation of CytC from the mitochondria to the cytoplasm is given by the following equations:


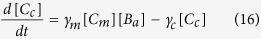



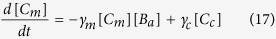


where *C*_*c*_ and *C*_*m*_ give the cytoplasmic and mitochondrial CytC concentrations; *B*_*a*_denotes the Bak oligomeric species forming the active pore (*a* = 4 for Model I, and *a* = 256 for Model II); and γ_m_ and γ_c_ give the rate constants for translocation of cytoplasmic and nuclear CytC. The total CytC concentration, *C*_*T*_ = [*C*_*c*_] + [*C*_*m*_], is taken to be a constant.

#### Numerical simulations

Simulations for both Models (Fig. 2B,C) were performed using numerical integration with a stiff ODE solver using the MATLAB SimBiology toolbox (Mathworks, Natick, MA). Initial conditions and parameter values for the simulations are shown in Table 1, and the simulation code is available as project files upon request.

#### Analytical solutions

To derive an analytical solution of the dynamics of the system, we first make the assumption that the timescales of Mcl-1 degradation are slow compared to that of Bak oligomerization and CytC translocation. This allows us to set the time derivatives of all differential equations, except (Eq. 4), to zero, and solve for the pseudo-equilibrium concentrations of all other species. Doing so, we find a single first-order equation that governs the dynamics of this system:


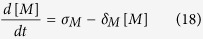


which has the solution:


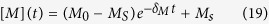


where *M*_*0*_ and *M*_*s*_ are the initial and final levels of free Mcl-1 in this system. We also find that the steady-state concentrations of


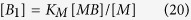



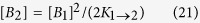



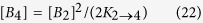


where the dissociation constants are 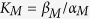, 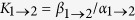, with higher order dissociation constants and equilibrium expressions being similarly defined. If we further assume that only a small percentage of Bak is oligomerized during apoptosis induction, as observed experimentally^35^, such that most Bak is either bound to Mcl-1, or unbound in monomeric form, we get that:


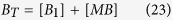


By combining this equation with (Eq. 20), we get the following relationship between free Mcl-1 level and Bak monomer level:


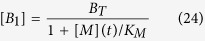


Next, by solving for the fraction of CytC in the cytoplasm using (Eqs 16, 17), we can show that:


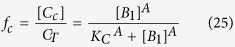


where *A* is the number of subunits in the active pore complex (*A* = 4 for Model I, and *A* = 256 for Model II), and *K*_*C*_ is the critical value of [*B*_1_], at which the level of cytoplasmic CytC is half its maximal value. It can be shown that this critical value is a function of the dissociation constants of the oligomerization reaction (*α* and *β*), and also the forward and back rate constants for CytC translocation (*γ*_*c*_ and *γ*_*m*_).

Based on these approximations, we now derive analytical expressions for: 1) time to the MOMP transition *T*_*c*_, which we define to be the time at which there is half-maximal CytC translocation to the cytoplasm, and 2) sharpness of the MOMP transition *ΔT*, which we define to be the time between 1/10 and 1/2 maximal CytC translocation. To derive these quantities, we first use (Eqs 24, 25) to derive the concentrations of free Mcl-1, where CytC translocation is 1/10 and 1/2 maximal:


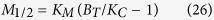



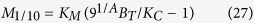


Next, by substituting (Eq. 26) in (Eq. 19), we find that the time delay until the MOMP transition is given by:


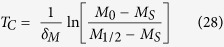


This expression reveals that the time delay preceding MOMP transition is set by the rate of Mcl-1 protein degradation *δ*_*M*_, and also scales logarithmically with initial levels of Mcl-1 protein. Next, we can show that the sharpness in the timing of the MOMP switch itself satisfies the following inequality:


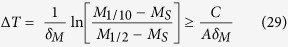


where C = In(9) ≈ 2.2. Note that lower bound for the MOMP switch timing is independent of the initial Mcl-1 level *M*_*0*_, as to be expected, and scales inversely with the size of the Bak pore. It is also independent of the detailed rate constants of the dimerization reactions, suggesting that this result does not depend on the exact reaction scheme, through which active pores are assembled.

### Computational derivation of mitotic survival curves based on the Bcl-2 network model

Using the above kinetic model (Eqs 8, 9, 10, 11, 12, 13, 14, 15, 16, 17, 18, 19, 20, 21, 22), we now generate predicted curves for the fraction of surviving mitotic cells over time, which will then be used to fit curves from experimental data. To better account for the cell death kinetics in the four cell lines studied, we first incorporate interactions between Bak and Bcl-xL by adding to the model the following rate equation:


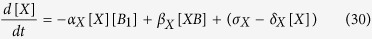


where [*X*] is the concentration of Bcl-xL. By solving for the steady state of this system using (Eq. 30) and the above equations, we obtain the following equation for the time evolution of free Bak monomer:


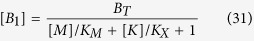


As the rest of the equations describing Bak oligomerization remain unchanged, the relationship between the concentration of Bak monomer and the cytoplasmic CytC fraction remain the same:


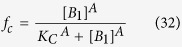


Taking into account our results (Fig. 2C), we now take the size of the Bak apoptotic pore to be very large, such that *A*→∞. This allows calculation of the time of apoptotic induction *t*_c_, which occurs when [*B*_1_] = *K*_*c*_. By incorporating this equation into (Eq. 31), we now get that:


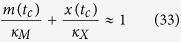


where *m*(t) and *x*(t) represent the ratios of free Mcl-1 and Bcl-xL levels to Bak level respectively, 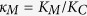 and 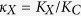. Here, we have further made the assumption that the total Bak level far exceeds the level required for apoptosis induction (*B*_*T*_ ≫ *K*_*c*_), consistent with the observation that most cells can switch into an apoptotic state upon induction. Now, by combining these equations with decay curves for Mcl-1 and Bcl-xL:


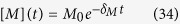



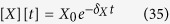


we can determine the time to death *t*_c_ as a function of variables that can be measured experimentally, including initial Mcl-1 and Bcl-xL levels, their decay time constants, and Bak levels (Fig. 3A). As the remaining biochemical parameters 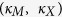 are properties of the proteins themselves, they would be expected to be invariant across different cells and cell lines; thus, they effectively provide scaling factors to the measured concentrations.

To obtain mitotic survival curves, defined as the fraction of mitotically arrested cells that remain alive as a function of time, we now use the results above to obtain the death time distribution for a cell population with variable levels of Mcl-1, Bcl-xL and Bak. This approach takes the hypothesis that non-genetic variability in protein levels constitutes the dominant source of variability in the timing of apoptosis decision, as previously proposed^24^. Following previous work^36^, we assume that variability of the protein level is well-described by a log-normal distribution, such that:


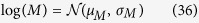



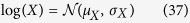



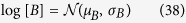


where *M, X* and *B* are random variables of the concentrations Mcl-1, Bcl-xL and Bak, and 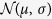 represents a normal distribution with mean *μ* and variance *σ*. Using these distributions, and the analytical expressions above (Eqs 33, 34, 35), we now numerically calculate the probability distribution of death times for a cell population as a function of parameters, which can then be integrated to obtain this cumulative distribution function:


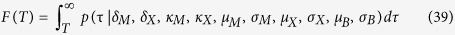


This function gives the mitotic survival curve, i.e. the probability that a cell survives for time *T* during mitotic arrest (Fig. 3A, center).

### Calculating the corrected mitotic survival curves from experimental single cell data

After cells enter drug-induced mitotic arrest, they can either die by apoptosis, or exit from mitosis, a competing processes that can cause cells to escape death^5^. Therefore, to properly compare mitotic survival curves derived from our kinetic model with experimental data (Eq. 39), we need to first generate experimental survival curves, where competing effects of mitotic exit are eliminated. In order to do so, we first take mitotic death and mitotic exit to be independent events, an assumption that has been validated experimentally^37^. This allows us to write the following simple model to describe the cell number dynamics during mitotic arrest:


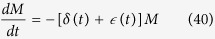



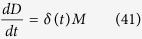



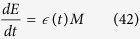


Here *M*, *D* and *E* represent the number of mitotically arrest cells, dead cells and exited cells, respectively; and *δ* (*t*) and 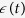 represent the rates of death and exit, respectively, which can change in a time-dependent manner during mitotic arrest, but vary independently. To estimate *δ* (*t*) from experimental data, we directly measure *M*, *D* and *E*, from time-lapse single-cell imaging data, then invert (Eqs 41 and 42) to obtain:


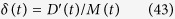


We can now use the following relations to obtain the mitotic survival curve, i.e. the probability that a cell survives in mitotic arrest, given that it does not undergo mitotic exit. We start with this equation:


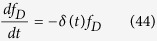


And plug in equation (43) to get:


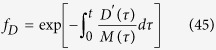


Similarly, we can derive a mitotic exit curve, which is the probability that a cell exits from mitotic arrest, given that it stays alive:


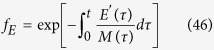


Using Eqs 43 and 44, we then generated mitotic survival and exit curves for all cell lines studied both under control conditions and upon knockdown of apoptotic regulator proteins. The calculated mitotic survival curves were then used for fitting to the model curves (see below); mitotic exit curves were not further used, but were found to be invariant to knockdown of Mcl-1 or Bcl-xL for all cell-lines examined, consistent with our previous finding that mitotic death and exit are independent cellular processes^37^.

### Constrained fitting of mitotic survival data to the kinetic model

Mitotic survival curves generated by model simulations were fitted to experimental data using a multi-step constrained least-squares procedure. We first determined initial conditions for least-squares optimization by performing a large random search of parameter space separately for each cell line, using bounds set by the experimental measurements of Mcl-1 half-life and average Mcl-1, Bcl-xL and Bak levels (Fig. 1C,D). From this search, we then chose a reduced set of points in this parameter space that gave the lowest value for the sum-squared of error, and then performed subsequent least-squares minimization using a pattern search procedure, using each point as an initial starting condition. This procedure gave rise to a set of optimized solutions for each individual cell line, accounting for mitotic survival kinetics under both control and protein knockdown conditions. From the set of solutions obtained from this procedure, a subset of solutions were found to recapitulate the observed cell-line differences in Bcl-2 protein levels from replicate western blot data. Mitotic survival curves for these fits, as well as corresponding parameters for all the cell lines are displayed in Fig. 3B–D.

## Additional Information

**How to cite this article**: Kueh, H. Y. *et al*. A simplified Bcl-2 network model reveals quantitative determinants of cell-to-cell variation in sensitivity to anti-mitotic chemotherapeutics. *Sci. Rep.* **6**, 36585; doi: 10.1038/srep36585 (2016).

**Publisher’s note:** Springer Nature remains neutral with regard to jurisdictional claims in published maps and institutional affiliations.

## Supplementary Material

Supplementary Information

Supplementary Information

## Figures and Tables

**Table 1:** Initial conditions and parameters for numerical simulations of apoptosis induction in mitotic arrest.

Initial conditions/parameter values	Value	Units	Description
[*B*_*1*_], [*B*_*2*_], [*B*_*4*_], [*B*_*8*_], [*B*_*16*_], [*B*_*32*_], [*B*_*64*_], [*B*_*128*_], [*B*_*256*_]	0.0	nM	Bak oligomeric species
[*M*]	3000.0	nM	Free Mcl-1
[*MB*]	1000.0	nM	Mcl-1/Bak complex
[*C*_*m*_]	1000.0	nM	Mitochondrial CytC
[*C*_*c*_]	0.0	nM	Cytoplasmic CytC
*σ*_*M*_	5 × 10^−6^	nM/s	Mcl-1 synthesis rate in mitosis
*δ*_*M*_	1.3 × 10^−4^	1/s	Mcl-1 degradation rate in mitosis
*α*_*M*_	0.05	1/nM/s	Bak/Mcl-1 association rate
*β*_*M*_	0.5	1/s	Bak/Mcl-1 dissociation rate
*γ*_*m*_	5000.0 (Model I) 0.5 (Model II)	1/nM/s	CytC cytoplasmic translocation rate
*γ*_*C*_	0.5	1/s	CytC mitochondrial translocation rate
*α*_*1→2*_	0.05	1/nM/s	Bak dimerization rate
*β*_*1→2*_	500.0	1/s	Bak dimer dissociation rate
*α*_*2→4*_	0.05	1/nM/s	Bak tetramer association rate
*β*_*2→4*_	0.05	1/s	Bak tetramer disassociation rate
*α*_*i→j*_	1	1/nM/s	Association rates of all other oligomers
*β*_*i→j*_	1	1/s	Dissociation rates of all other oligomers
