# Supplementary material for: A simplified Bcl-2 network model reveals quantitative determinants of cell-to-cell variation in sensitivity to anti-mitotic chemotherapeutics
Source: Sci Rep. 2016 Nov 4;6:36585. doi: 10.1038/srep36585 (PMC5095668; doi:10.1038/srep36585)
Supplement: Supplementary Information [file srep36585-s2.doc]

**A simplified Bcl-2 network model reveals quantitative determinants of cell-to-cell variation in sensitivity to anti-mitotic chemotherapeutics**

Hao Yuan Kueh^1, 2, 4^, Yanting Zhu^2, 3^ and Jue Shi ^2,3*^

^1^Division of Biology, California Institute of Technology, Pasadena, CA 91125, USA

^2^Center for Quantitative Systems Biology, Hong Kong Baptist University

^3^Department of Physics and Department of Biology, Hong Kong Baptist University, Hong Kong, China

^4^Current address: Department of Bioengineering, University of Washington, Seattle, WA 98195, USA

**Supplementary Information**

**Supplementary movie SM1**. Fluorescent time-lapse movie of the MOMP reporter, IMS-RP, in a representative HeLa cell treated with 1 μM K5I. Time is indicated in unit of day:hour:minute. Cell was imaged every 10 minutes. The cell entered mitotic arrest at time frame of 17:40, i.e., 17 hours and 40 minutes after drug addition. The abrupt change of punctate to smooth distribution of the MOMP reporter fluorescence occurred during mitotic arrest between time frames of 1:04:20 and 1:04:30.
